# Data on solute carrier transporter genes of a threatened Himalayan fish species – *Schizothorax richardsonii*

**DOI:** 10.1016/j.dib.2019.103712

**Published:** 2019-03-07

**Authors:** Ashoktaru Barat, Prabhati Kumari Sahoo, Rohit Kumar, Chirag Goel, C. Siva, Shahnawaz Ali

**Affiliations:** a ICAR-Central Institute of Freshwater Aquaculture, Bhubaneswar 751002, Odisha, India; b Advanced Centre for Treatment, Research and Education in Cancer (ACTREC), Kharghar, Navi Mumbai 410210, Maharashtra, India; c ICMR – Model Rural Health Research Unit, Civil Hospital, Haroli, 177220 Una, Himachal Pradesh, India; d ICAR-Directorate of Coldwater Fisheries Research, Bhimtal, 263136 Nainital, Uttarakhand, India

**Keywords:** RNA-Seq, Molecular characterization, Cellular transporters

## Abstract

The Snowtrout, *Schizothorax richardsonii*, is a vulnerable fish species found in different rivers and rivulets of the Himalayan region. The species is also a suitable poikilotherm to study the low-temperature tolerance as it dwells well at a temperature range of 5–20 °C. The solute carrier (SLC) group of membrane transport proteins play an integral role in cellular acclimation response. The present RNA sequencing was done to identify solute carrier transporter which are the major gene cascades responsible for transport of sugars, amino acids, oligonucleotides, ions, drugs, etc. to and from the cell organelles. A reference transcriptome database was created from liver tissue of *Schizothorax richardsonii* through RNA sequencing on Illumina HiSeq 2000 platform. The sequences were annotated and characterized under various solute carrier families in the species. So far, 113 transcripts were identified as solute carrier transporter genes categorized under 13 different families. This data will be useful for many researchers working on gene cloning and differential expression of solute carriers.

**Table:** Specifications table

Subject area	*Biology*
More specific subject area	*Transcriptome*
Type of data	*Transcriptome sequences*
How data was acquired	*RNA sequencing using Illumina HiSeq 2000 platform*
Data format	*Raw .fasta sequence in text file and table*
Experimental factors	*Liver samples of both control and heat shocked individuals*
Experimental features	*De novo assembly, GO annotation, Gene identification using Blastx tool*
Data source location	*Bhubaneswar, India*
Data accessibility	*Data is being provided with this article*
*Related research article*	*Barat A., Sahoo P.K., Kumar R., Goel C., Singh A.K. (2016) Transcriptional response to heat shock in liver of snow trout (Schizothorax richardsonii)—a vulnerable Himalayan Cyprinid fish, Funct. Integr. Genomics. 16 203–213.*https://doi.org/10.1007/s10142-016-0477-0

**Table:** 

**Value of the data** • The dataset is a readily available reference of solute carrier transporter genes mined from the liver transcriptome of a non-model fish. • These will be helpful for researchers working on the same species or closely related species particularly on transporters, ion channels, etc. • Useful for differential gene expression and phylogenetic analysis with evolutionary and comparative genomics studies.

## 1 Data

The raw RNA-Seq data is available on NCBI GENBANK (SRA acc. No. SRX643306). The present dataset is extracted (annotated and characterized as solute carrier transporter) from the *de novo* assembly of transcriptome data of *Schizothorax richardsonii.* Nucleotide sequences of all solute carrier genes identified in the transcriptome of *S. richardsonii* is provided in ‘*.txt*’ file format.

## 2 Experimental design, materials and methods

The details of sample collection, experimental design, RNA isolation, and sequencing were reported earlier [1]. RNA sequence data of *Schizothorax richardsonii* (Bio-project accession no PRJNA253239) were retrieved from NCBI GenBank (https://www.ncbi.nlm.nih.gov/bioproject/PRJNA253239). The raw reads were subjected to quality check using Fast QC v.0.11.8 followed by trimming using Fastx toolkit V.0.0.13. The trimmed and cleaned reads of both (SRR1503433 and SRR1552917) were pooled for *de novo* assembly using CLC Genomics Workbench (QIAGEN Bioinformatics, USA). The contigs so obtained were further assembled into transcripts using CAP3 [2]. The coding sequences of assembled transcripts were identified using TransDecoder v.5.5.0 (https://github.com/TransDecoder/TransDecoder/releases) on default parameters. The CDS generated through TransDecoder were matched for functional annotations using nonredundant BLASTx tool of NCBI GenBank (https://blast.ncbi.nlm.nih.gov). The gene identified as solute carrier transporters were extracted from complete transcriptome assembly using in-house pearl script (Table 1).

**Table 1:** Details of solute carrier families observed in the liver transcriptome of *Schizothorax richardsonii.*

Contig ID	SLC	Gene ID	Gene name	GeneBank	BLASTx e value
**Inorganic anion/cation transport**					
1062810	slc4	slc4a1a	band 3 anion transport protein	NP_938152.1	0.0
1066568		Slc4a2a	anion exchange protein 2	NP_001032314.1	0.0
1068006		Slc4a2b	anion exchanger	NP_001107912.1	0.0
1072522		Slc4a7	Predicted: Sodium bicarbonate cotransporter 3	XP_009290880.1	0.0
1036206	Slc9	Slc9a3r1	Na (+)/H (+) exchange regulatory cofactor NHE-RF1	NP_997872.2	2e-135
1069006	Slc12	Slc12a2	potassium/chloride transporters	NP_001002080.1	0.0
1070924		Slc12a4	Predicted: potassium/chloride transporters	XP_691291.2	0.0
1046148		Slc12a6	potassium/chloride transporters	XP_020792176.1	6e-69
1068568		Slc12a8	potassium/chloride transporters	NP_001121749.1	0.0
1017415		Slc12a9	potassium/chloride transporters	NP_001122020.1	3e-91
1069036	Slc20	Slc20a1a	sodium-dependent phosphate transporter	NP_998344.1	0.0
1059836		Slc20a1b	sodium-dependent phosphate transporter 1-B	NP_997753.1	2e-162
1063094	Slc26	Slc26a1	sulphate anion transporter 1	NP_001074136.1	0.0
1054148		Slc26a2	Predicted: sulfate transporter	XP_020359215.1	4e-178
1068556		Slc26a5	prestin	NP_958881.1	0.0
1059638		Slc26a11	sodium-independent sulfate anion transporter	NP_956061.1	0.0
**Amino acid and oligopeptide transport**					
1061674	Slc1	Slc1a3a	excitatory amino acid transporter 1	NP_997805.1	0.0
1035372	Slc3	Slc3a2b	amino acid transporter heavy chain	NP_958922.2	4e-108
1057762	Slc7	Slc7a1	PREDICTED: high affinity cationic amino acid transporter 1 isoform	XP_683623.4	0.0
1068818		Slc7a3	cationic amino acid transporter, y + system	NP_001007330.2	0.0
1063888		Slc7a6	Y + L amino acid transporter 2	NP_001018310.1	0.0
1018741		Slc7a9	PREDICTED: B (0,+)-type amino acid transporter 1	XP_005169080.1	3e-77
1061224	Slc17	Slc17a5	Sialin [Danio rerio] anion/sugar transporter	NP_001070195.1	0.0
1062834		Slc17a9b	anion/sugar transporter	NP_001002635.1	0.0
1069738	Slc36	Slc36a1	proton-coupled amino acid transporter 1	XP_687732.4	0.0
1069074	Slc38	Slc38a2	sodium-coupled neutral amino acid transporter 2	NP_001038569.1	0.0
1070630		Slc38a3	sodium-coupled neutral amino acid transporter 5	NP_001002648.1	0.0
1067430		Slc38a4	sodium-coupled neutral amino acid transporter 4	NP_001005944.2	0.0
1034094		Slc38a6	probable sodium-coupled neutral amino acid transporter 6	NP_001018308.1	6e-144
1067684		Slc38a7	putative sodium-coupled neutral amino acid transporter 7	NP_001003648.1	0.0
1022612	Slc43	Slc43a1a	amino acid system L transporter	NP_001314915.1	2e-132
1002903		Slc43a1b	large neutral amino acids transporter small subunit 3	NP_001076469.1	2e-87
1013829		Slc43a2a	large neutral amino acids transporter small subunit 4	NP_956545.1	2e-61
972610		Slc43a2b	large neutral amino acids transporter small subunit 4	NP_001008585.1	4e-82
1059342		Slc43a3b	solute carrier family 43 member 3	NP_001035011.1	0.0
**Transport of glucose and other sugars**					
1066294	Slc2	Slc2a1	solute carrier family 2, facilitated glucose transporter member 1-like	XP_020346873.1	0.0
1009559		Slc2a2	glucose transporter 2	KC513421.2	1e-106
1068576		Slc2a8	solute carrier family 2, facilitated glucose transporter member 6	NP_997963.1	0.0
1062118		Slc2a12	solute carrier family 2, facilitated glucose transporter member 12	NP_956832.1	0.0
1039720	Slc5	Slc5a2	sodium/glucose cotransporter 2	NP_998091.1	9e-88
1044578	Slc50	Slc50a1	sugar transporter SWEET1	NP_001012515.1	2e-115
**Transport of bile salts and organic anions**					
1045170	Slc10	Slc10a3	SLC10A3-like protein, partial	KJ777502.1	5e-105
1067754	Slc13	Slc13a5	Di- and tricarboxylate transporter [Carbohydrate transport and metabolism]	NP_001136038.1	0.0
**Monocarboxylate transporter**					
1065692	Slc16	Slc16a2	thyroid hormone transporter	NP_001245159.1	0.0
1056880		Slc16a3	monocarboxylate transporter 4	NP_997873.1	0.0
1071962		Slc16a4	monocarboxylate transporter 5	NP_001074068.2	0.0
1061320		Slc16a7	monocarboxylate transporter 2 like	XP_004070979.1	0.0
1016957		Slc16a8	Uncharacterized protein	NP_997797.1	1e-53
1067292		Slc16a9a	solute carrier family 16, member 9a	NP_956704.1	0.0
1021626	Slc47	Slc47a2	multidrug and toxin extrusion protein 1	NP_001073648.1	8e-94
**Metalion transport**					
1048352	Slc30	Slc30a1a	zinc transporter 1	NP_957173.1	0.0
1069498		Slc30a5	zinc transporter 5	NP_001002322.1	0.0
1040510		Slc30a7	Co/Zn/Cd efflux system component	AB987984.1	0.0
1003631	Slc39	Slc39a1	zinc transporter ZIP1	XP_020353560.1	1e-14
1039056		Slc39a4	zinc transporter ZIP4	NP_001124249.1	1e-28
1044232		Slc39a6	zinc transporter ZIP6	NP_001001591.1	6e-76
1043306		Slc39a9	zinc transporter ZIP9	NP_001013558.1	0.0
1067228		Slc39a10	ZIP Zinc transporter	AB988007.1	0.0
1059932		Slc39a13	zinc transporter ZIP13	NP_001005306.3	0.0
1045216	Slc41	Slc41a1	Divalent cation transporter	FQ310506.3	0.0
**Transport of urea, neurotransmitters and biogenic amines, ammonium and choline**					
1069812	Slc6	Slc6a13	neurotransmitter transporter	NP_001004533.1	0.0
987653	Slc18	Slc18b1	MFS-type transporter	NP_001017841.1	7e-44
1051398	Slc22	Slc22a2	organic cation transporter	NP_998315.1	0.0
1031164	Slc22	Slc22a18	organic cation transporter	NP_001032462.1	3e-102
1051684	Slc44	Slc44a4	choline transporter-like protein 4	NP_956707.1	0.0
**Transport of vitamins and cofactors**					
1035792	Slc23	Slc23a1	nucleobase transporters	NP_001166970.1	4e-174
1064658	Slc33	Slc33a1	acetyl-coenzyme A transporter 1	NP_957402.1	2e-136
1062610	Slc46	Slc46a1	proton-coupled folate transporter	NP_956579.1	0.0
997095	Slc52	Slc52a2	riboflavin transporter 1	NP_955950.1	8e-87
**Nucleoside/nucleotide transport**					
1065114	Slc29	Slc29a1a	Equilibrative nucleoside transporter 1	NP_001025348.1	0.0
1031960	Slc35	Slc35b1	solute carrier family 35 member B1	NP_001004583.1	4e-57
1063694		Slc35b2	adenosine 3′-phospho 5′-phosphosulfate transporter 1 precursor	NP_991198.1	0.0
1055528		Slc35b3	adenosine 3′-phospho 5′-phosphosulfate transporter 2	NP_001035084.1	0.0
1047578		Slc35b4	UDP-xylose and UDP-*N*-acetylglucosamine Transporter	NP_997817.2	0.0
1045786		Slc35c1	GDP-fucose transporter 1	NP_001008590.1	0.0
1027680		Slc35c2	ovarian cancer overexpressed 1: Triose-phosphate Transporter family	NP_997808.1	2e-123
1055762		Slc35e1	solute carrier family 35 member E1	NP_998239.1	1e-179
1013693		Slc35e2	solute carrier family 35 member E2-like	XP_020362107.1	5e-98
1049536		Slc35e4	solute carrier family 35 member E4	NP_001017857.1	8e-171
1064524		Slc35f2	solute carrier family 35, member F2	NP_001070024.1	0.0
**Transport of fatty acids, prostaglandins and steroid sulphates**					
1053548	Slc27	Slc27a1a	long-chain fatty acid transport protein 1	NP_001013555.1	0.0
994313		Slc27a1b	long-chain-acyl-CoA synthetase	NP_001070716.1	1e-84
1020140		Slc27a2	long-chain-acyl-CoA synthetase	NP_001020470.1	5e-44
1053812		Slc27a4	long-chain fatty acid transport protein 4	NP_001017737.1	0.0
1055764		Slc27a6	long-chain fatty acid transport protein 6 precursor	NP_001070854.1	0.0
**Transport of fatty acids, prostaglandins and steroid sulphates, thyroid sulphates**					
	nil				
**Heme transport**					
1054106	Slc48	Slc48a16	heme transporter hrg1-A	NP_956300.1	7e-85
**Transport across mitochondrial membranes**					
907636	Slc25	Slc25a3b	mitochondrial carrier; phosphate carrier	BC067565.1	7e-21
1044412		Slc25a5	ADP/ATP transporter on adenylate translocase	NP_775354.1	0.0
1054268		Slc25a6	adenine nucleotide translocator), ADP/ATP translocase 3	NP_989562.2	0.0
1025440		Slc25a10	dicarboxylate transporter,	NP_957466.1	2e-98
1072488		Slc25a12	mitochondrial carrier, Aralar 1	NP_997947.1	0.0
1048712		Slc25a14	brain mitochondrial carrier protein 1	NP_956458.1	0.0
1047404		Slc25a16	mitochondrial carrier protein/graves disease carrier protein	NP_991112.1	0.0
1035454		Slc25a20	carnitine/acylcarnitine translocase	NP_957153.1	4e-173
1063614		Slc25a21	mitochondrial oxodicarboxylate carrier	NP_001070100.1	0.0
1062104		Slc25a22	mitochondrial glutamate carrier	NP_998573.1	3e-169
1037430		Slc25a25	calcium-binding mitochondrial carrier proteinSCaMC-2-A	NP_998422.1	4e-144
987757		Slc25a26	adenosylmethionine mitochondrial carrier protein	NP_001025314.1	2e-99
979606		Slc25a27	mitochondrial uncoupling protein 4	NP_956635.1	3e-54
1067474		Slc25a28	mitoferrin-2	NP_998284.2	3e-119
1065884		Slc25a29	mitochondrial carnitine/acylcarnitine carrier protein CACL	NP_001025408.1	2e-155
1006145		Slc25a32a	mitochondrial folate transporter/carrier	NP_956550.1	3e-113
1061138		Slc25a32b	mitochondrial folate transporter/carrier	NP_001013354.1	0.0
1041964		Slc25a33	solute carrier family 25 member 33	NP_998322.1	4e-151
1022370		Slc25a36a	solute carrier family 25 member 36-A	NP_001002667.1	2e-129
1045510		Slc25a37	mitoferrin-1	NP_001035060.1	4e-101
888952		Slc25a38a	solute carrier family 25 member 38A	NP_001070070.1	1e-27
1026578		Slc25a39	solute carrier family 25 member 39	NP_956780.1	2e-159
1058676		Slc25a40	solute carrier family 25 member 40	NP_001002360.1	0.0
1046498		Slc25a42	mitochondrial coenzyme A transporter SLC25A42	NP_001038918.1	0.0
1064802		Slc25a43	solute carrier family 25 member 43	NP_001004497.2	0.0
1058484		Slc25a44a	solute carrier family 25, member 44 a	NP_001007351.1	0.0
1019095		Slc25a47b	hepatocellular carcinoma down-regulated mitochondrial carrier homolog B	NP_001083050.1	1e-91

## References

[1] Barat A., Sahoo P.K., Kumar R., Goel C., Singh A.K. (2016). Transcriptional response to heat shock in liver of snow trout (*Schizothorax richardsonii*)—a vulnerable Himalayan Cyprinid fish. Funct. Integr. Genomic..

[2] Huang X., Madan A. (1999). CAP3: a DNA sequence assembly program. Genome Res..

